# Transgenic Mouse Models Enabling Photolabeling of Individual Neurons *In Vivo*

**DOI:** 10.1371/journal.pone.0062132

**Published:** 2013-04-23

**Authors:** Manuel Peter, Brice Bathellier, Bruno Fontinha, Pinelopi Pliota, Wulf Haubensak, Simon Rumpel

**Affiliations:** Research Institute of Molecular Pathology (IMP), Vienna, Austria; Instituto de Neurociencias de Alicante UMH-CSIC, Spain

## Abstract

One of the biggest tasks in neuroscience is to explain activity patterns of individual neurons during behavior by their cellular characteristics and their connectivity within the neuronal network. To greatly facilitate linking *in vivo* experiments with a more detailed molecular or physiological analysis *in vitro*, we have generated and characterized genetically modified mice expressing photoactivatable GFP (PA-GFP) that allow conditional photolabeling of individual neurons. Repeated photolabeling at the soma reveals basic morphological features due to diffusion of activated PA-GFP into the dendrites. Neurons photolabeled *in vivo* can be re-identified in acute brain slices and targeted for electrophysiological recordings. We demonstrate the advantages of PA-GFP expressing mice by the correlation of *in vivo* firing rates of individual neurons with their expression levels of the immediate early gene *c-fos*. Generally, the mouse models described in this study enable the combination of various analytical approaches to characterize living cells, also beyond the neurosciences.

## Introduction

Patterns of action potentials in neuronal circuits are believed to be the neurobiological correlate of psychological phenomena such as percepts, thoughts or decisions. Recent advances in electrophysiological recording techniques or optical calcium imaging techniques *in vivo* have yielded much information about firing patterns in populations of neurons in response to particular sensory stimuli or in the context of behavioral tasks [1], [2]. However, it is still difficult to explain or even to predict the emergence of the various activity patterns that can be observed in a given neuronal population. One major step towards this goal is to gain additional information about a neuron that goes beyond the description of its firing patterns. Typically, information about cell-intrinsic properties such as the expression profile of specific receptors or voltage-gated channels is difficult to obtain from neurons recorded *in vivo*. However, such information could be valuable for the identification of the cell type [3]. In addition, in most cases the connectivity of the neuron under observation remains unknown and it is unclear what specific inputs it actually receives. Encouragingly, in the last years a number of approaches have been established in mammalian model organisms to overcome some of these limitations. There are several approaches to identify the cell type of the neuron recorded *in vivo*: Besides classic cell-labeling techniques through a recording electrode [4], [5], genetic approaches in which reporter genes are expressed under the control of specific promoters are increasingly used to identify a particular cell type [6]. When optical recording techniques are used, the expression of a fluorescent protein can identify a subset of neurons belonging to the same cell type [7], [8]. For electrical recording techniques cell type specific expression of Channelrhodopsin allows selective light-induced firing and thus can provide information about the identity of the recorded cell [9].

Several novel technical developments also allow a better understanding of the connectivity of neurons recorded *in vivo.* A combination of recordings with large-scale anatomical reconstruction of neuronal circuits using electron microscopy has recently been demonstrated [10]. However, it needs to be considered that many functional aspects of anatomically identified connections still remain unknown and that a significant effort is needed for the reconstruction of even relatively small tissue samples. Complementary to this method large scale calcium imaging in the mouse visual cortex *in vivo* has been combined with random paired patch-clamp recordings in brain slices and post-hoc re-alignment of patched neurons [11], [12]. This elegant approach works best when there is a high density of neurons of interest and useful recordings can be obtained with a high probability. Further approaches include the usage of transsynaptically transported viruses for labeling interconnected populations [13] or identification of specific projection neurons by antidromic stimulation [9]. In summary, there have been encouraging efforts to combine *in vivo* recordings with a further analysis of the recorded neurons.

Here, we generated and characterized genetic mouse models that can be optimally combined with optical recording techniques and that allow the selection and photolabeling of individual neurons for subsequent analysis of their morphology, cell type or connectivity. Photolabeling techniques, as previously demonstrated, can overcome some of the shortcomings of the previously mentioned approaches and has its particular advantages if the neurons of interest are sparse and if the neurons are preferably maintained alive for further analysis [14], [15]. Our strategy relies on photoactivatable fluorescent proteins that allow conditional labeling of cells upon irradiation with light of a particular wavelength. A number of switchable proteins have been described over the past years [16]. One of them, Kaede, has been recently used to generate a transgenic mouse [17]. However, Kaede is not reliably photoswitchable using two-photon excitation (own observation, [18], [19], but see [20]). Two-photon illumination, in contrast to one-photon illumination, allows selective excitation of a diffraction limited spot within scattering tissue. This is essential for high-resolution photolabeling in the living animal where closely located cells can show dramatic differences in functional properties. We generated and characterized three mouse lines expressing photoactivatable GFP (PA-GFP, [21]), that can be optimally used for optical recordings of neuronal activity and that allow photolabeling of individual neurons for subsequent analysis of their morphology, expression pattern, cell type or connectivity. These mouse lines allow the selective photolabeling of individual neurons for hours to days and *in vivo* monitoring of neuronal activity can be readily combined with subsequent *in vitro* electrophysiology and a morphological or an immunohistochemical characterization of these neurons.

## Materials and Methods

All animal experiments were performed in accordance with the Austrian laboratory animal law guidelines for animal research and had been approved by the Viennese Magistratsabteilung 58 (Approval #: M58/02182/2007/11; M58/02063/2008/8 and M58/002220/2011/9).

### Cell Culture

#### Hek293 cells

PA-GFP [21], PS-CFPII [22], PamCherry1 [23], Dendra2 [24], Kaede [25] and KikGR [26] fused to a NLS were cloned in the pCMV-MCS vector (Stratagene) using standard molecular cloning techniques. Hek293 cells were grown to 80% confluency and transfected with Lipofectamin 2000 (Invitrogen 11668-019) and the plasmid DNA containing the PA-FP following the manufacturers protocol. 24 h later the cells were fixed for 5 min with 4% Paraformaldehyde (PFA).

#### Cortical neurons

Cortical neurons were isolated from E17 C57bl6 mouse embryos. Cortices were removed and digested with 0.25% Trypsin (GIBCO 15050-065) for 3 min at 37°C to get single cells. Neurons were resuspended in BME medium (GIBCO 41010-026) supplemented with 1% Penicillin-Streptomycin (GIBCO 15140-122), 1% L-Glutamin 200 mM (GIBCO 25030-024), 1% Insulin-Transferrin-Selenium-A Supplement (GIBCO 51300-044), 0.6% Glucose solution (40%) and 10% FBS (GIBCO 10500-064) and seeded at a density of 10000 cells on Poly-L-Lysine Coated Coverslips (BD 354085). Neurons were incubated for 1 h at 37°C, 5% CO2 and then the medium was changed to Neurobasal medium (GIBCO 21103) supplemented with 5% B27 supplement (GIBCO 17504-044), 0.5% GlutaMAX (GIBCO 35050) and 1% Penicillin-Streptomycin (GIBCO 15140-122). Neurons were incubated at 37°C and 5% CO_2_ for 7 days and then transfected with Lipofectamin 2000 following the manufacturers protocol. 72 h after transfection neurons were used for the photoactivation experiments.

### *In vitro* Imaging

Hek293 photoactivation experiments were done using an Ultima *in vivo* multiphoton microscopy system (Prairie Technologies) with a 20×0,95 numerical aperture objective lens (Olympus) and a Ti:Sapphire multiphoton laser (Coherent). To define the best imaging wavelength for an activated PA-FP, cells were first photolabeled and then imaged at wavelength ranging from 850 nm–950 nm. To test if the PA-FP can be switched using 2-photon illumination ROIs were activated at different wavelength ranging from 720 nm–940 nm and imaged afterwards to see if a significant fluorescence increase was induced. To test the fluorescence increase induced by photoactivation ROIs were scanned multiple times (1–128 times), an image was taken at the optimal imaging wavelength and the fluorescence increase was quantified using ImageJ.

Photoactivation experiments in primary cortical cultures were performed on an upright LSM 780 confocal laser scanning microscope (Carl Zeiss MicroImaging GmbH, Germany). Photoactivation at the soma was done using a 405 nm diode. Green (PA-GFP) and red (tdTomato) fluorescence signals were captured simultaneously by using the 488 and 561 nm laser lines. The settings for the photoactivation as well as the settings for the imaging of the neurons were kept the same throughout the experiments. Fluorescence of tdTomato was only mildly affected by the photoactivation procedure (∼6% loss). Images were quantified using a custom written script in Matlab (Mathworks): The red channel at T-1 min. was thresholded to obtain a mask defining the outline of the photolabeled neurons. For the given time points the ratio images of the red and green channels were computed, all values outside the mask were clipped to zero. The ratio at the soma was computed by averaging all ratio pixel values within a user-defined region of interest.

### Histology

#### PA-GFP staining

8–12 week old mice were sacrificed, the brain removed and fixed in 4% PFA at 4°C overnight. On the next day the brains were dehydrated in a graded alcohol series, embedded in paraffin and cut to 2 µm slices. PA-GFP staining was performed on a Discovery XT (Ventana Medical Systems) machine. The rabbit polyclonal anti GFP antibody (Abcam ab290) was used at a concentration of 1∶1000. PA-GFP was visualized using a secondary biotynilated goat anti rabbit antibody (Dako, E 0432) in combination with steptavidin-HRP using DAB as a substrate. The slices were scanned on a Mirax Scan (Carl Zeiss MicroImaging GmbH, Germany) slide scanner. For the R26 PA-GFP::NLS mice the slices were incubated for 2 h at RT with the secondary antibody Dye-Light 549 goat anti rabbit IgG (Thermo Scientific 35507) dilution 1∶1000 in PBS containing 5% normal goat serum and 0.1% Triton-X 100.

#### GABA/CamKII/NeuN staining

Mice were transcardially perfused with 20 ml PBS containing 10 U/ml Heparin (Sigma H3393) and 20 ml 4% PFA. The brains were removed and post-fixed over night in 4%PFA at 4°C. They were cut on a vibratome (VT-1000, Leica) to 70 µm thick slices and incubated for 2 h at RT in PBS containing 10% normal goat serum (Jackson Immuno research 005-000-121) and 1% Triton-X 100 (Sigma-Aldrich T8787). Subsequently, brain slices were washed with PBS and incubated with the primary antibody for GABA dilution 1∶1000 (Sigma A2052) or CamKII dilution 1∶75 (Abcam ab52476) in PBS containing 5% normal goat serum and 0.1% Triton-X 100 at 4°C overnight. On the next day the incubation was continued for 1 h at RT. Afterwards the slices were washed 3×10 min with PBS and incubated with the secondary antibody Dye-Light 549 goat anti rabbit IgG (Thermo Scientific 35507) dilution 1∶1000 in PBS containing 5% normal goat serum and 0.1% Triton-X 100 at RT for 2 h. To counter stain for NeuN positive cells a primary NeuN antibody (Millipore MAB377) was conjugated with the Zenon Labelling kit (Molecular Probes Z-25013) following the manufacturers protocol and added at a dilution of 1∶100 to the secondary antibody mixture. After 2 h the slices were washed 3×10 min with PBS, post fixed for 15 min with 4% PFA, washed 2×10 min with PBS and mounted on cover slips. The slides were imaged on a LSM700 confocal laser scanning microscope (Carl Zeiss MicroImaging GmbH, Germany).

#### Fos staining

Directly after the *in vivo* Ca imaging session mice were transcardially perfused with 20 ml PBS containing 10 U/ml Heparin (Sigma H3393) and 20 ml 4% PFA. The brains were removed and post-fixed for 45 min in 4%PFA at 4°C. Next, brains were embedded in low melting Agarose (Sigma A9793) and cut parallel to the imaging plane into 70 µm slices on a vibratome (VT-1000, Leica). Slices were incubated for 2 h at RT in PBS containing 10% normal goat serum (Jackson Immuno research 005-000-121) and 1% Triton-X 100 (Sigma-Aldrich T8787). Afterwards brain slices were washed with PBS and incubated with the primary c-fos antibody at a dilution of 1∶1000 (Santa Cruz Biotechnology, sc-52) in PBS containing 5% normal goat serum and 0.1% Triton-X 100 at 4°C overnight. On the next day the incubation was continued for 1 h at RT. Subsequently, the slices were washed 3×10 min with PBS and incubated with the secondary Alexa Fluor 647 goat anti–rabbit IgG Antibody (Molecular Probes A-21244) dilution 1∶1000 in PBS containing 5% normal goat serum and 0.1% Triton-X 100 at RT for 2 h. Then the slices were washed 3×10 min with PBS and mounted on cover slips. The slides were imaged on a LSM510 Axiovert 200M confocal laser scanning microscope (Carl Zeiss MicroImaging GmbH, Germany).

### Generation of Genetically Modified Mice

#### Thy1.2-PA-GFP::NLS lines

The Thy1.2 vector as described by [27] was generously provided by Pico Caroni (Friedrich Miescher Institute for Biomedical Research, Basel). The PA-GFP::NLS fusion was cloned into the *Xho*I site of the Thy1.2 plasmid and the expression construct was recovered by *Pvu*I and *Eco*RI digestion. To generate transgenic mice the expression construct was injected in fertilized oocytes using standard techniques. The embryos were obtained from crosses between (C57BL6/J and CBA) F1 hybrids. Transgenic founders were identified using PCR with the following primers: Thy1fw (CTACCAGCTGGCTGACCTGTAG) which binds to the Thy1 sequence and PAGFPRV (CTTGTCGGCCATGATATAGACGTTG) which binds to the PA-GFP sequence. Positive founders were back-crossed to C57BL6/J mice and the expression pattern of the transgene was analyzed using immunohistochemistry as described above.

#### R26 PA-GFP::NLS mice

The pROSA26-1 targeting plasmid [28] was used to generate the PA-GFP::NLS knock in mice. First the PA-GFP::NLS and the WPRE sequence were cloned into the pCCALL2 plasmid [29] which contains the CAGGS promoter to generate pCCALL2-PA-GFP::NLS. The construct was cut using *Asc*I and *Asis*I and the resulting fragment was cloned into a modified pROSA26-1 plasmid to generate the final targeting construct. The construct contains the 5′ and 3′ homology arms, the CAGGS promoter, a betaGeo cassette flanked by loxP sites, the PA-GFP::NLS transgene and a WPRE sequence. The construct was linearized using *Acc*65I and electroporated into A9 129/B6 F1 hybrid ES cells, which were established from blastocysts isolated from C57Bl6 females mated to 129 males [30] using standard techniques. Neomycine resistant clones were screened by southern blot analysis with a probe which binds to the 5′ arm. Positive ES cell clones were injected into C57BL6/J blastozysts. Highly chimeric mice were bread with C57BL6 mice. Successful targeting of the R26 locus was verified by PCR and southern blotting.

### Targeted Patch-clamp Whole-cell Recordings from Photolabeled Neurons

#### Slice preparation

Mice were sacrificed by quick cervical dislocation, decapitated and the brain rapidly removed from the skull. The brain was immersed (approximately for 1 minute) in ice-cold oxygenated (95% O2/5% CO2) dissection solution containing (in mM) 110 choline chloride, 25 NaHCO3, 1.25 NaH2PO4, 2.5 KCl, 0.5 CaCl2, 7 MgCl2, 11.6 ascorbic acid, 3.1 pyruvic acid and 25 D-glucose (final pH ≈ 7.4 after infusion with carbogen (95% O2/5% CO2). Acute coronal whole-brain slices (300 µm-thick) were made using a vibratome (Leica VT1200S, Germany). Slices were then transferred to a resting chamber filled with standard artificial cerebrospinal fluid (ACSF) composed of (in mM) 118 NaCl, 2.5 KCl, 26.5 NaHCO3, 1 NaH2PO4, 1 NgCl2, 2 CaCl2 and 20 D-glucose, aerated with 95% O2/5% CO2, for 30 minutes at a temperature of 33°C, and subsequently maintained at room temperature throughout the experiments.

#### Electrophysiology

Fluorescent neurons were identified in the brain slice using an Olympus BX51WI (Olympus, Japan) upright epifluorescence microscope equipped with a 100-W power range mercury short-arc lamp (USHIO, Tokyo, Japan) and with infrared (IR) video microscopy and differential contrast optics. Whole-cell patch-clamp recordings in current-clamp mode were acquired from the somata of the identified fluorescence neurons with Multiclamp 700B amplifiers (Axon Instruments, Molecular Devices, Foster City, CA). Patch pipettes were pulled from borosilicate glass (2.0 mm outer and 1.16 mm inner diameter glass, Warner Instruments) on a Flaming/Brown micropipette puller (Sutter Instrument, Novato, CA), yielding a final resistance of 3–5 MΩ. The pipette intracellular solution contained (in mM) 130 K-gluconate, 5 KCl, 2.5 MgCl2, 10 HEPES, 0.6 EGTA, 4 Na2ATP, 0.4 Na3GTP and 10 Na2-phosphocreatine (pH = 7.25 adjusted with KOH; 290 mOsm). To characterize the pattern of neuronal action potential firing, a series of 500 ms current pulses were applied in 20 pA steps, from −40 to 340 pA. Electrophysiological data was low-pass filtered using the 10 and 3 kHz four-pole Bessel filter, sampled at 10 kHz (Digidata 1440A, Axon Instruments) and collected using pClamp10 software (Molecular Devices, Inc., USA). Offline analysis of intrinsic neuronal properties was made using the data analysis software Clampfit 10.2 (Molecular Devices, Inc., USA). All the recordings were made at room temperature.

### Photolabeling using Optical Fibers

Mice were anesthetized with isofluorane and an optical fiber (Thor Labs BFL37-200) with an inner diameter of 200 µm and a NA of 0.37 was stereotaxically inserted into the perirhinal cortex (Coordinates: −1.06 mm posterior from Bregma, 4 mm lateral from midline, 3 mm ventral from pial surface). Photoactivation of PA-GFP was performed for 5 min at constant illumination at 405 nm with an output power at the tip of the fiber between 5.5 and 7.5 mW. Immediately afterwards, the mice were transcardially perfused with 20 ml PBS containing 10 U/ml Heparin (Sigma H3393) and 20 ml 4% PFA. The brains were removed and post-fixed for 2 h in 4%PFA at 4°C. They were cut on a vibratome (VT-1000, Leica) to 100 µm thick slices and incubated at RT for 20 min with SYTO60 (Molecular Probes S11342) diluted 1∶20000 in PBS. Afterwards, the slices were washed with PBS, mounted on cover slips and imaged on an LSM780 confocal laser scanning microscope (Carl Zeiss MicroImaging GmbH, Germany). To measure the area of photoconverted PA-GFP, the image of the slice that contained the fiber tract was identified and background fluorescence subtracted and fluorescence normalized to peak levels. For each slice fluorescence was measured along a line following the longitudinal axis of the fiber. The horizontal spread was measured perpendicular to the first line 125 µm below the tip.

### *In vivo* Imaging

#### Surgery

Thy1.2-PA-GFP::NLS or R26 PA-GFP::NLS mice in the age of 8–12 weeks where used for the imaging experiments. To obtain optical access to the auditory cortex a small imaging window was implanted over the auditory cortex as described elsewhere [31].

#### *In vivo* imaging

*In vivo* calcium imaging and photoactivation was done using an Ultima *in vivo* multiphoton microscopy system (Prairie Technologies) with a 20× objective lens (XLUMPlan Fl, n.a. = 0.95, Olympus) and a Ti: Sapphire multiphoton laser (Coherent). Mice were anesthetized with Isofluorane and photoactivation was done at 750 nm and imaging of the photolabeled neurons at 950 nm.

#### Time course

Positions of single neurons were identified based on their weak basal fluorescence. Next, a ROI was placed over the soma of the neurons and the neurons were photolabeled. Photolabeled neurons were revisited and imaged at different time points. For analysis, images were background subtracted and the fluorescence of the individual neurons was normalized to the fluorescence level directly measured after photolabeling.

#### Reactivation

Single neurons were photolabeled and revisited after 24 h. After measuring their fluorescence they were photolabeled and again revisited after 24 hours. For analysis, images were background subtracted and the fluorescence was normalized to the fluorescence level measured directly after photolabeling.

#### Filling of neurons

Single neurons were consecutively photolabeled 3 times at a time interval of 15minutes. Subsequently, image stacks were taken from the photolabeled neurons and the dendritic morphology was reconstructed using IMARIS software (Bitplane, Switzerland).

#### *In vivo* Ca imaging

A wide craniotomy (∼1×2 mm) was performed above the right auditory cortex under isoflurane anesthesia (1.5 to 2%). Dye preparation and injection were done according to standard procedure [32]. The calcium sensitive dyes Rhod2-AM or Oregon Green BAPTA 1 (OGB1) were dissolved in DMSO and 20% Pluronic acid to a concentration of 2.5 mM or 10 mM respectively. This stock solution was diluted 1/10 into the pipette solution (150 mM NaCl, 2.5 mM KCl and 10 mM HEPES) and pressure ejected (0.7 bar: 10 pulses of 10 sec) in the brain through a thin glass pipette (∼5 MΩ tip resistance) using a Femtojet (Eppendorf). Injections were performed at several loci to increase the coverage of the auditory cortex. The craniotomy was then closed with a thin cover glass and sealed with dental cement (Ortho-Jet, Lang Dental). A metal post was also implanted on the head for fixation of the animal in the imaging apparatus and kept under light isoflurane anesthesia (1%).

Fields of stained neurons were imaged using a two-photon microscope (Ultima IV, Prairie Technologies) equipped with a 20× objective (XLUMPlan Fl, n.a. = 0.95, Olympus). Rhod2-AM and OGB1 were excited at 900 nm and 950 nm respectively using a pulsed laser (Chameleon Ultra, Coherent). In all experiments, the field of view was set to be 200×200 µm. Neurons were detected on an initial image of the field of view using a semi-automated method implemented in a custom-built Matlab (The Mathworks) software. The image (512×512 pixel) was band-pass filtered (Gaussian: high cut 6.5 pixels, low cut 30 pixels) and local maxima of intensity were detected as putative neurons. Detection errors and astrocytes were removed by the user based on their morphology. The line scan trajectory was computed to cover all user-confirmed neurons with a minimal displacement (approximate solution of the travelling salesman problem obtained by a genetic algorithm). A ∼2 µm-wide cross corresponding to an added travel length of ∼10 µm was drawn on each neuron to increase the dwell time of the line scan on neurons with respect to neuropile. This allowed us to increase the signal to noise ratio of neuronal recordings. The fluorescence from any given neuron was the average signal from all segments of the line scan that were within 3.5 µm from its center. Line scans rate was between 33 to 25 lines/seconds, depending on the number of recorded neurons.

All recordings consisted of 8 blocks of 15 seconds separated by a minimum of two seconds. The normalized change in calcium fluorescence *ΔF/F* was computed in each block. The selection of most or least active neurons was based on the following analysis. To gain temporal precision, the real time course of the neuronal firing rate was evaluated by deconvolution [33] of a single exponential kernel with a single time constant τ = 1.3 sec corresponding to the typical decay time of calcium transient for both Rhod-2 and OGB1. The mean population activity was computed as the deconvolved calcium signals averaged across all simultaneously recorded neurons. Bursts of population activity were detected as peaks of the mean population activity that were 3 standard deviations above the mean. A 250 ms time bin centered on the peak of each burst was defined and the relative change of *ΔF/F* during this time bin was computed for each neuron as a surrogate of its spiking activity. The mean activity of each neuron was computed as the mean *ΔF/F* change across all detected bursts. This measure was used to rank the neurons and select the 20% most or least active neurons for photoactivation. An alternative method based on a template matching algorithm, independent of burst detection, was also used to estimate spontaneous activity levels of individual neurons, and gave qualitatively similar results.

### Analysis of Fos Expression Levels

PA-GFP labeled neurons were identified in the fixed slices and a z-stack containing the neurons was taken on an LSM510 Axiovert 200M confocal laser scanning microscope (Carl Zeiss MicroImaging GmbH, Germany). Additionally, they were registered to a stack that was taken *in vivo* after the labeling and the scan line was superimposed. This procedure confirmed the high fidelity of the photolabeling procedure. On the image plane containing the photolabeled neurons the fluorescence of all Fos positive cells was measured: for each cell a standardized, round ROI was manually positioned on the soma and the mean fluorescence calculated using ImageJ. The values for a given image plane (47–231 cells) were standardized using their z-score to compensate for possible differences in global staining intensity. The z-score was calculated by subtracting for each cell’s fluorescence the population mean and dividing by the population standard deviation. If a given photolabeled neuron did not give a detectable Fos signal, the ROI for this cell was positioned using the PA-GFP signal.

## Results

### Selection of a Photactivatable Fluorescent Protein for in vivo Expression

As a first step towards the generation of a mouse model that would allow photo-tagging of individual neurons in the living brain, we characterized several PA-FPs. The optimal PA-FP would show low cytotoxicity, low basal fluorescence, high fluorescence after activation and is effectively activatable using two-photon excitation. We expressed six paFPs in cultured HEK293 cells (PA-GFP [21], PS-CFPII [24], PAmCherry [23], Kaede [25], KikGR [26], Dendra2 [34]) and characterized their one and two-photon activation properties. We found that only a subset of the characterized paFPs was efficiently activatable using two-photon excitation (Table 1). Among those, PA-GFP showed the strongest change in fluorescence following activation (Figure S1). We therefore selected PA-GFP for the following experiments.

**Table 1:** Functional characterization of various photoactiavatable/photoswitchable proteins.

PA-FP	single photon activation	2-photon activation	activation wl (nm)	imaging wl (nm)	fluorescence increase (x fold)
**PA-GFP**	nf/green	+	740	950	86
**PS-CFPII**	cyan/green	+	740	940	43
**PAmCherry**	nf/red	+	800–880	950	3,2
**Dendra2**	green/red	−			
**Kaede**	green/red	−			
**KikGR**	green/red	−			

**Figure 1:**
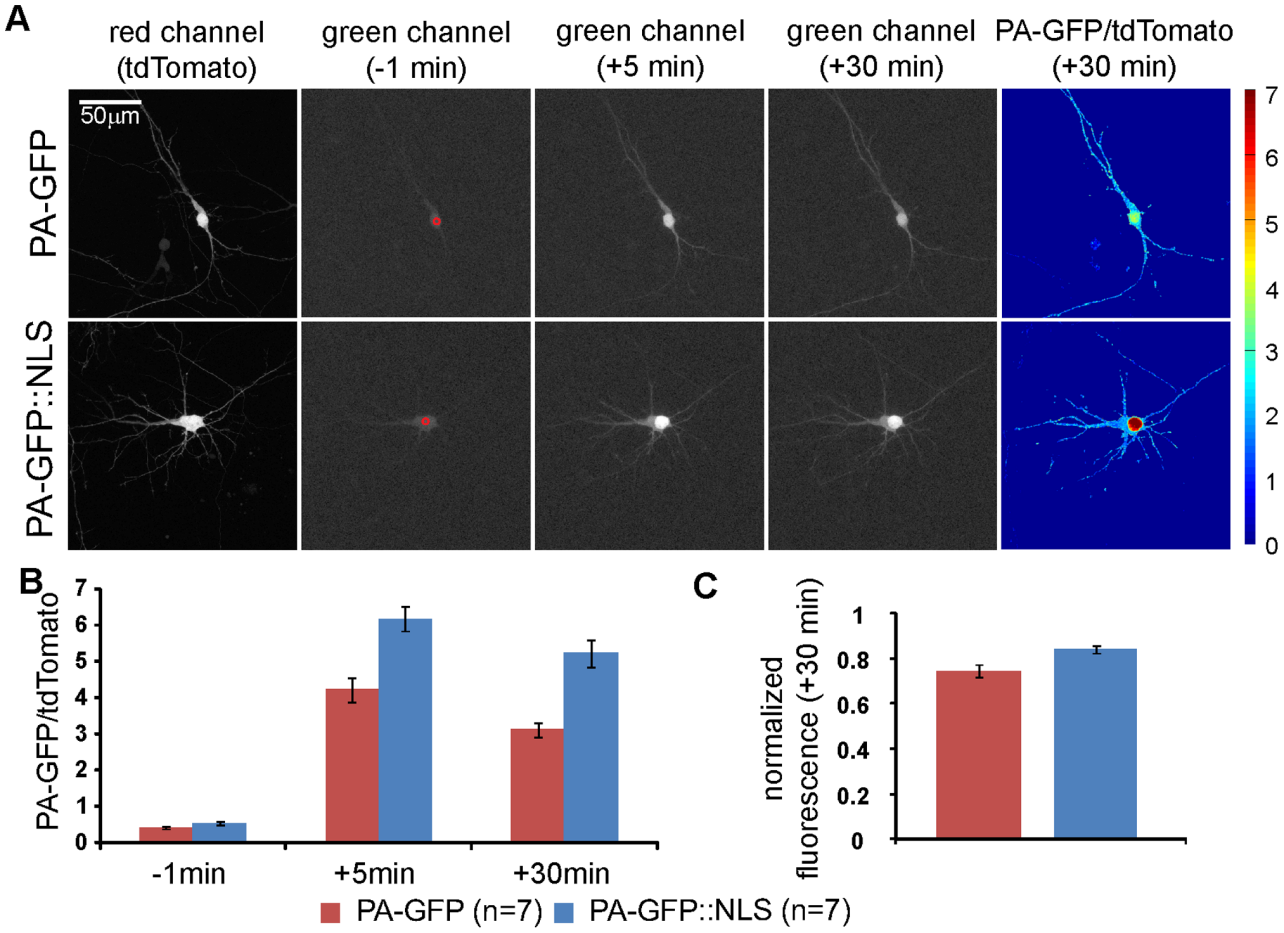
The nuclear localization signal enriches PA-GFP at the soma. A: PA-GFP or PA-GFP::NLS were co-expressed with tdTomato in primary cortical neuronal cultures. PA-GFP was photoactivated at the soma (red circle). Images were taken before (−1 min), directly after (+5 min) and 30 min (+30 min) after photolabeling. Representative images in the red and green fluorescence channel for PA-GFP and for PA-GFP::NLS expressing neurons are shown for several time points. Same gamma correction was applied to all monochrome images to visualize also relatively low fluorescence intensities of the dendrites. Pseudocolor images display ratio of intensities in green and red channels. B: The mean fluorescence ratio at the soma of green and red channels before, directly after and 30 min after photolabeling indicates higher labeling efficacy in neurons expressing PA-GFP::NLS. C: Decay of photolabel intensity at the soma quantified as normalized PA-GFP fluorescence at the soma 30 min after photolabeling.

Neurons are so tightly packed within the neuropil that individual dendrites and axons cannot be resolved with conventional light microscopy. Due to this limitation, photoactivation of PA-GFP would be predominantly targeted to the soma, which is big enough to be unambiguously identifiable *in vivo*. We reasoned that photolabeling of neurons is facilitated if PA-GFP was enriched at the soma. Therefore, we compared the efficiency of photolabeling in primary neuronal cultures expressing PA-GFP and PA-GFP fused to a nuclear localization sequence (PA-GFP::NLS) [35]. We co-expressed the constitutively fluorescent red fluorescent protein tdTomato [36] using a 2A strategy to identify transfected neurons before photolabeling and to normalize variations in expression levels [37]. We monitored the fluorescence intensity of PA-GFP at neuronal somata one minute before photolabeling, 5 min after and 30 min after photolabeling at the soma (Figure 1A). We found that neurons expressing PA-GFP::NLS showed significantly higher green/red fluorescence ratios at the soma after photolabeling as PA-GFP expressing neurons (−1 min: PA-GFP::NLS 0.54±0.05, PA-GFP 0.43±0.02, p = 0.09; +5 min: PA-GFP::NLS 6.18±0.35, PA-GFP 4.23±0.33, p<0.001; +30 min: PA-GFP::NLS 5.23±0.37, PA-GFP 3.12±0.21, p<0.001; Wilcoxon rank sum test; n = 7 neurons for PA-GFP::NLS and PA-GFP each; Figure 1B). For both constructs we observed a reduction in the green/red ratio in the measurements from 5 minutes to 30 minutes after photolabeling. This is likely due to diffusion of activated PA-GFP from the soma into the neurites, as their morphology became visible also in the green channel after photolabeling. This loss was less pronounced in neurons expressing PA-GFP::NLS (PA-GFP::NLS 84.3%±1.7%, PA-GFP 74.5%±3.0%, p<0.026; Wilcoxon rank sum test, Figure 1C), indicating that the NLS causes an enrichment, but not complete trapping of PA-GFP in the nucleus. Together, these findings indicate that the NLS can improve the photolabeling efficiency and we considered a PA-GFP::NLS fusion protein for the generation of transgenic mice.

### Generation of Genetically Modified Reporter Mice Expressing PA-GFP

**Figure 2:**
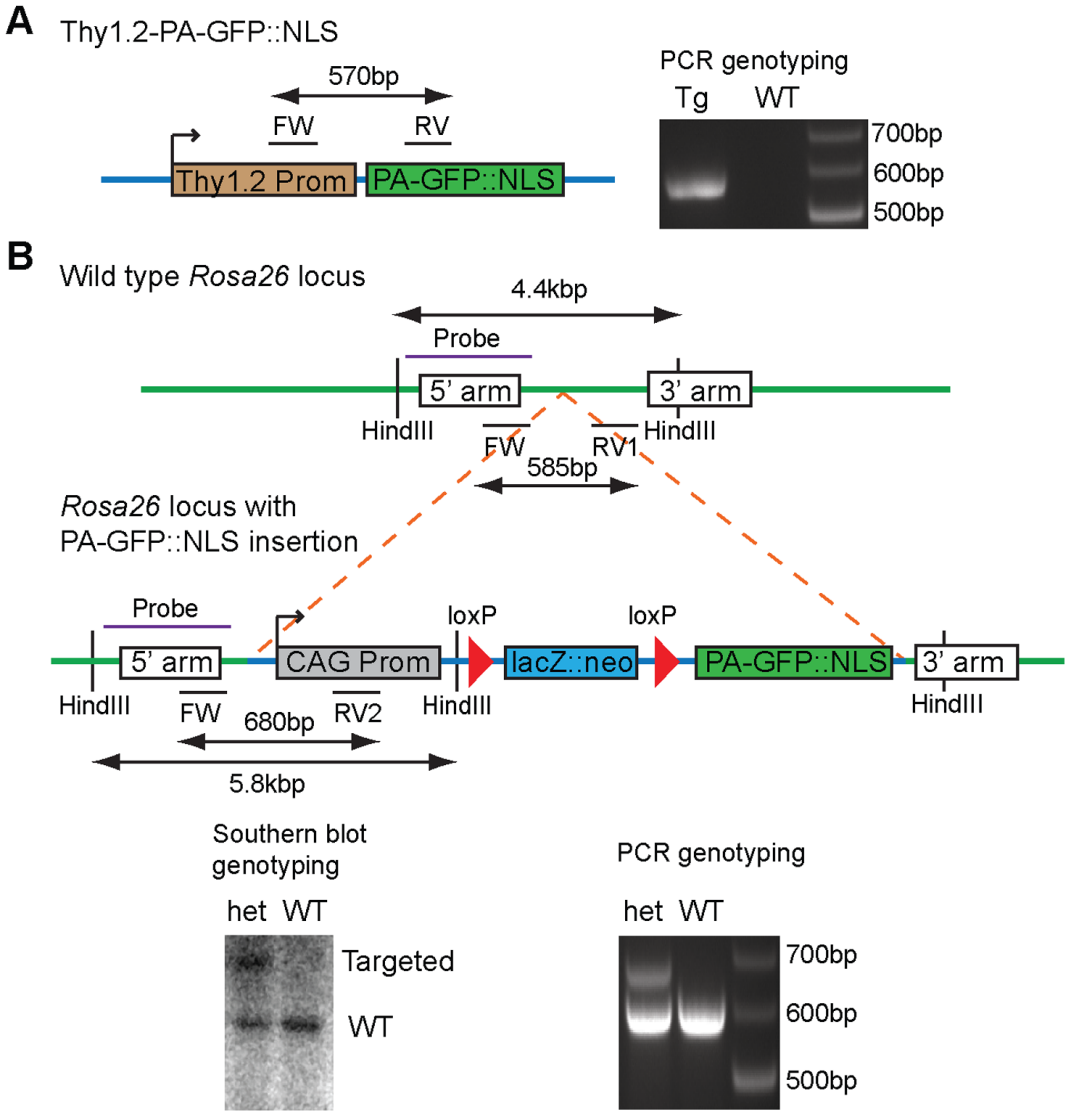
Genetic strategies for the generation of PA-GFP::NLS expressing mice. A: Schematic of the construct used for the generation of transgenic mice expressing PA-GFP::NLS under the control of the Thy1.2 promoter (left). ‘FW’, ‘RV’ indicates the location of binding sites of forward and reverse primers used for genotyping yielding a 570 bp PCR product. Representative image of a gel electrophoresis of products obtained from a genotyping PCR from a transgenic mouse (Tg) and a wild type (WT) mouse (right). B: Schematic diagram of the PA-GFP::NLS knock in strategy into the Rosa26 locus. After Cre-mediated recombination of loxP sites a stop cassette (lacZ::neo) is excised and leads to expression of PA-GFP::NLS under the control of the ubiquitous active CAGGS (CAG) promoter. ‘FW’, ‘RV1’ and ‘RV2’ indicates the location of binding sites of primers used for genotyping yielding a 585 bp or 680 bp PCR product for the wild type or knock in allele respectively. ‘Probe’ indicates the binding site for probe used for southern blot analysis after *Hind*III digestion of genomic DNA, resulting in the labeling of a 4.4 kb or 5.8 kb band in the southern blot. Representative southern blot shown for a wild type (WT) and heterozygous (het) mouse (bottom left). Representative image of a gel electrophoresis of products obtained from a genotyping PCR from a heterozygous mouse (het) and a wild type (WT) mouse (bottom right).

We used two strategies to generate genetically modified mice. We first used conventional transgenics, which relies on the random insertion of an expression construct in the genome (Figure 2A). The advantage of this system is that transgenic mice can be obtained in relatively short periods of time and can reach very high expression levels due to the insertion of multiple copies. However, individual transgenic founder lines typically show strong variability in their expression patterns despite the usage of the Thy1.2 promoter, which in the brain drives expression predominantly in neurons [27], [38]. As a complement, we also generated knock-in mice in which PA-GFP::NLS is expressed under control of the constitutively active CAGGS promoter from the targeted ROSA26 locus (line R26 PA-GFP::NLS; Figure 2B). Generally, this strategy leads to a broad expression in most cell types [29], [39]. However, as the targeting construct contains a stop-cassette that can be excised upon Cre-mediated recombination, crossing these reporter mice with Cre-driver lines can restrict expression of PA-GFP::NLS to genetically defined cell types.

**Figure 3:**
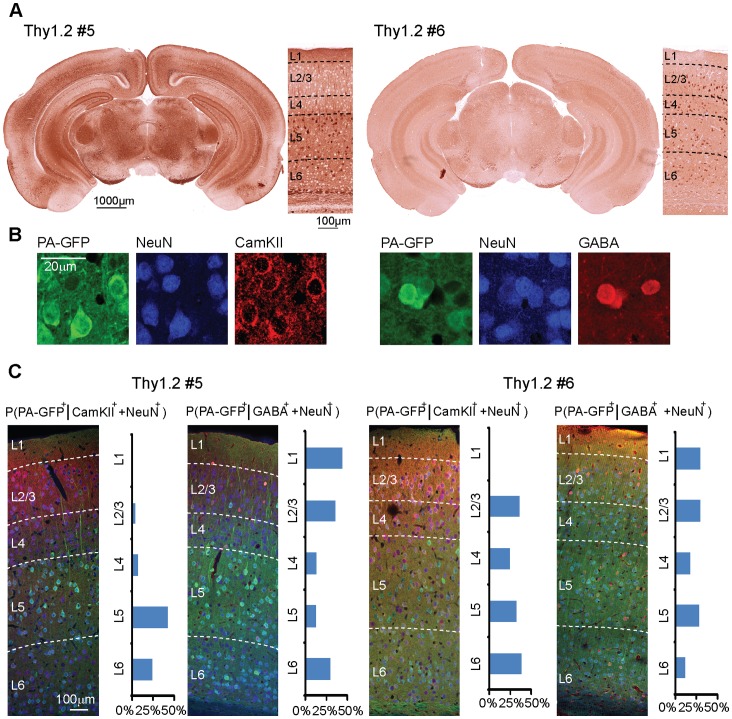
Expression patterns of PA-GFP::NLS in the transgenic mouse lines. A: Coronal brain sections of the Thy1.2#5 (left) and the Thy1.2#6 (right) lines immunohistochemically stained for PA-GFP. Neocortical layers are indicated by dashed lines in the higher-magnification images. B: Examples of sections stained for PA-GFP/NeuN/CamKII (left) and PA-GFP/NeuN/GABA (right). C: Quantification of cell type-specific expression of PA-GFP::NLS in the Thy1.2#5 (left) and the Thy1.2#6 (right) line. Neocortical layers are indicated by dashed lines on representative triple-stained coronal sections. Bar graphs represent fraction of NeuN and CamKII or NeuN and GABA double positive neurons that also express PA-GFP for all 6 cortical layers.

After generation of the transgenic lines we first characterized the expression patterns of PA-GFP::NLS in coronal brain sections using immunohistochemical detection of PA-GFP. We screened in total six Thy1.2 founder lines in which four showed significant expression in the brain. We focused on two of them: In mice of line Thy1.2#5 PA-GFP was strongly expressed in cortical layer 5 and fewer, but very strongly expressing neurons in layers 2/3 (n = 3 sections from 3 mice), representative section shown in Figure 3A). Mice of line Thy1.2#6 showed more evenly distributed expression across cortical layers, whereas the labeling intensity of individual neurons tended to be weaker as compared to line 5 (n = 3 sections from 3 mice, Figure 3A).

To further analyze the expression pattern of PA-GFP::NLS in the transgenic mice, we performed immunohistochemical detection of PA-GFP, the neuronal marker protein NeuN and as a third marker either CamKII for detection of excitatory neurons or GABA for detection of inhibitory neurons (Figure 3B). Coronal sections of the auditory cortex from three mice from lines Thy1.2#5 and Thy1.2#6 were stained and we quantified the fraction of NeuN and CamKII double positive neurons that were in addition GFP positive for the six cortical layers of both lines (Figure 3C). We found that in line Thy1.2#5 CamKII-positive neurons were predominantly expressing PA-GFP in layers 5 and 6, whereas GABA-positive neurons were mostly found in layers 1–3 and 6. In the Thy1.2#6 line CamKII-positive and GABA-positive neurons expressing PA-GFP were more evenly distributed across cortical layers.

To characterize the expression in R26 PA-GFP::NLS mice we analyzed mice that had been crossed to EMX1-Cre and Nestin-Cre driver lines. Both driver lines are expected to lead to broad PA-GFP::NLS expression in the brain [40], [41]. To assess possible background expression of PA-GFP::NLS despite the stop-cassette, we also analyzed the brains of R26 PA-GFP::NLS mice that do not express Cre. We found strong and broad expression of PA-GFP in brain sections from R26 PA-GFP::NLS×EMX1-Cre mice (n = 3 mice) whereas virtually no PA-GFP expression was detected in Cre-negative littermates (n = 3 mice, Figure S2A).

The mouse lines expressing PA-GFP are available from the Jackson Laboratory Repository with the JAX Stock No.****021069, 021070 and 021071.

### Functional Characterization of Mice Expressing PA-GFP::NLS

**Figure 4:**
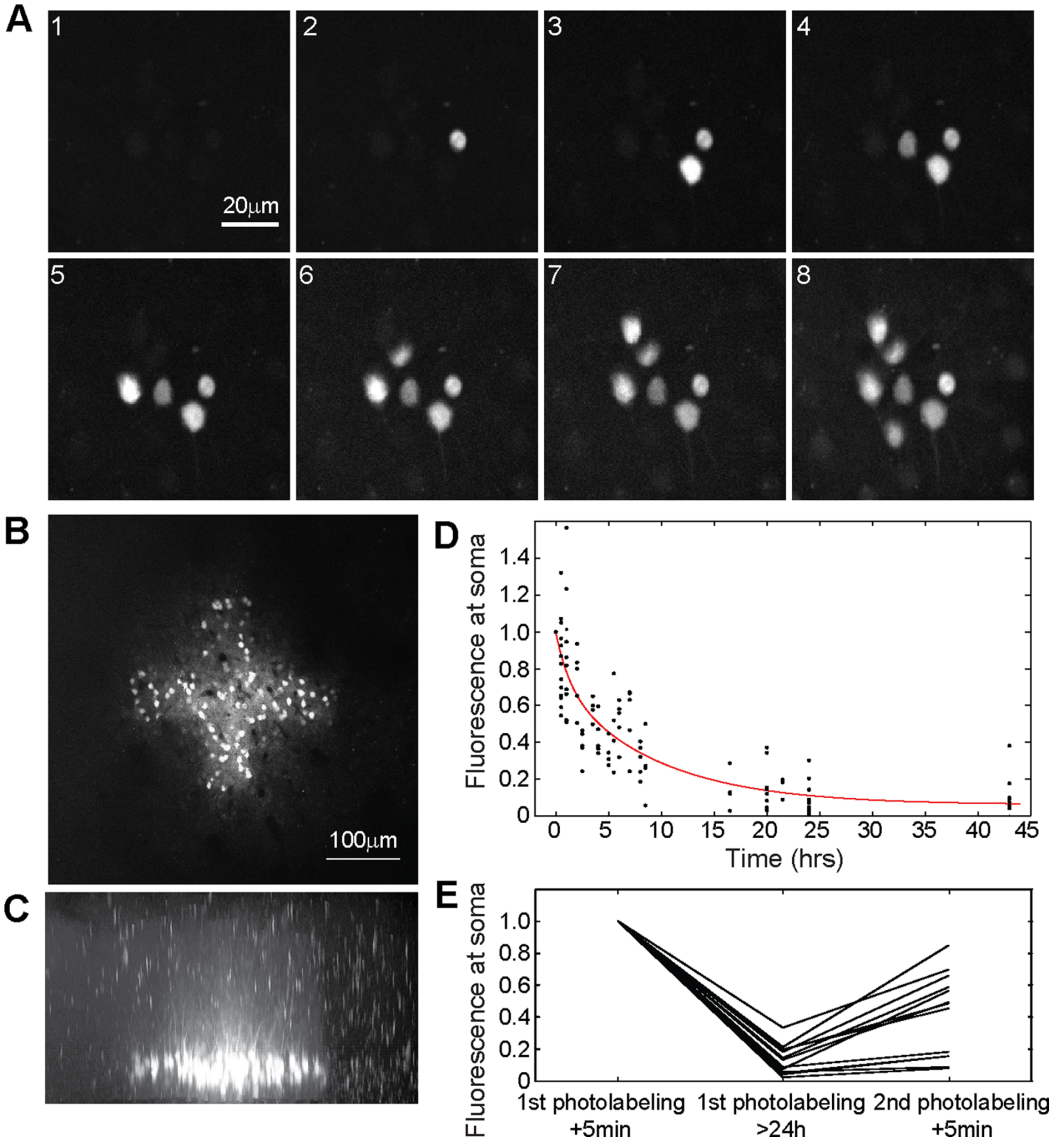
Photolabeling of neurons *in vivo*. A: Two-photon images of individual neurons consecutively photolabeled *in vivo*. Nearby neurons can be labeled with high precision. The time interval between photolabeling of single cells was ∼1 min. The imaging depth was ∼150 µm B: Bulk labeling of neurons in a cross shaped ROI. C: Side view of an image stack showing the same neurons displayed in (B). Note, only neurons in the plane of activation were photolabeled. D: Intensity of the photolabel at the soma at various time points after photolabeling. Points represent individual measurements (n = 4−9 measurements from 30 neurons). Red line indicates double exponential fit to the fluorescence decay. Individual photolabeled neurons can be found for more than one day. E: Re-labeling of previously photolabeled neurons. Lines represent normalized fluorescence intensity at the soma of individual neurons directly after initial photolabeling, after more than 24 hours and directly after second photolabeling.

**Figure 5:**
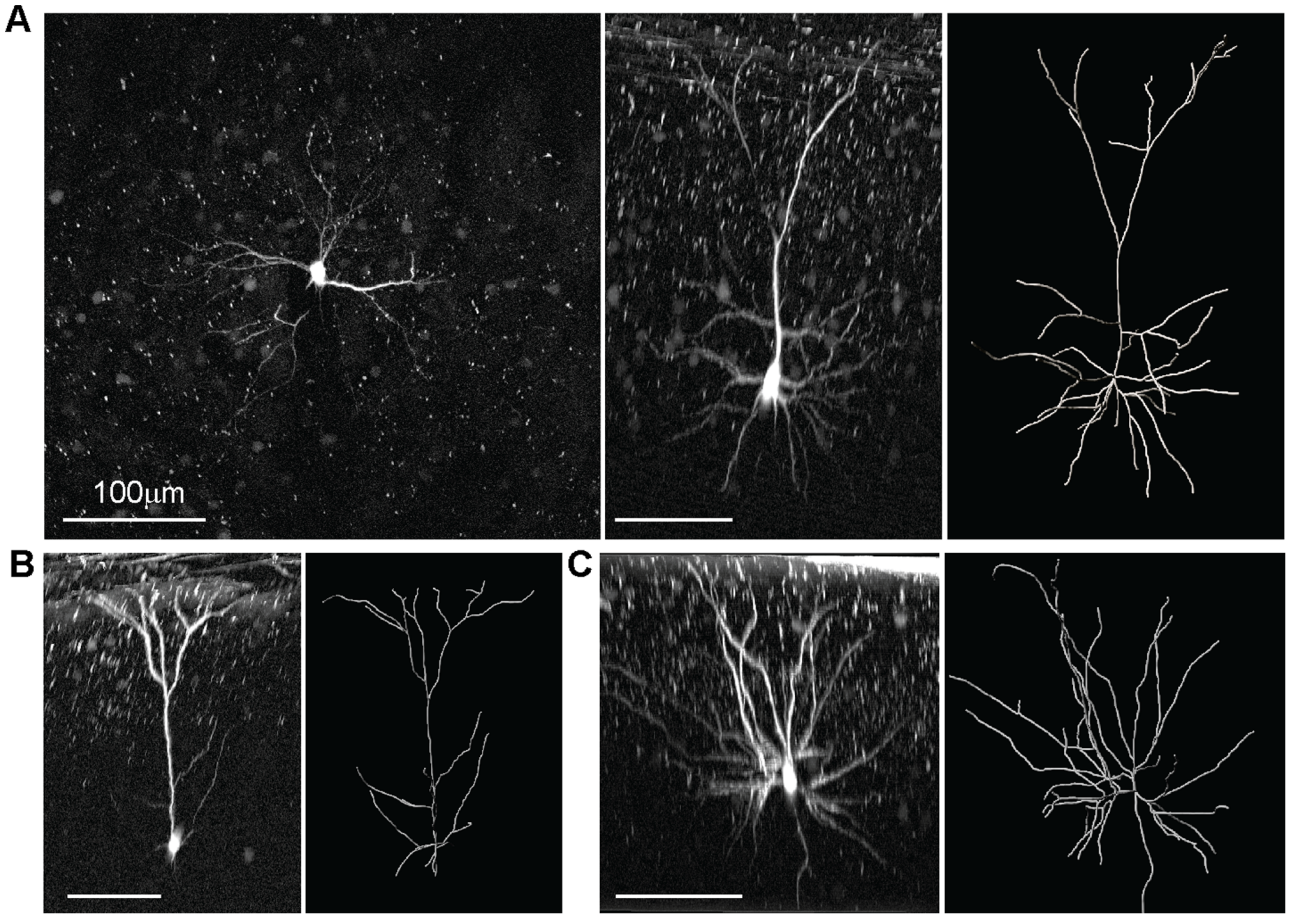
Photolabeling reveals dendritic morphology. A: Maximum intensity projection of an image stack taken *in vivo* of a previously photolabeled neuron (left). Side projection of the stack (middle) and *in silico* reconstruction of the neuron (right). B: Side projection of another putative pyramidal cell (left) and reconstruction (right). C: Side projection of an image stack taken *in vivo* of a putative interneuron (left) and reconstruction (right). All scale bars indicate 100 µm (see also Figure S3).

After confirmation of expression of PA-GFP::NLS in the cortex using histological methods, we were interested in testing the efficiency of photolabeling neurons in the living brain. Towards this end we implanted a small cranial glass window over the auditory cortex which provided us with chronic optical access to the brain [31]. Using two-photon laser scanning microscopy in anaesthetized mice, we were able to identify PA-GFP::NLS expressing cells based on basal fluorescence at very high laser intensity settings at 900–950 nm excitation wavelength. In all three lines we were able to readily photolabel neurons at a depth of typically 100–300 µm below the dura after brief excitation at 720–750 nm at the soma (Thy1.2#6: Figure 4A–C; Thy1.2#5: Figure 5A; R26 PA-GFP::NLS: Figure S2B). The two-photon approach provided us with sufficient resolution to label single, nearby neurons (Figure 4A) without labeling neurons above or below the focal plane (Figure 4B, C). This would not have been achievable using conventional one-photon excitation.

How long does the photolabel persist? We again implanted mice with cranial windows and photolabeled individual neurons in cortical layers 2/3. We re-visited the neurons at various time intervals up to two days and acquired images with identical power and detection settings (line Thy1.2#5, n = 3 mice, 30 neurons, each imaged at 4–9 time points after photolabeling). We found that the fluorescence intensity at the soma decays significantly over the time course of hours to days (Figure 4D). Based on our previous observations in primary neuronal cultures we reasoned that two processes could have a major influence on the decay: at a shorter time scale the diffusion of the activated PA-GFP::NLS from the soma into the dendrites and at a longer time scale the turnover of the fluorescent protein itself [42]. The decay of fluorescence over time *f(t)* could be well approximated by the following double exponential function:



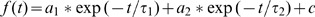



The fitted parameters were: *a*_1_ = 0.27; 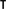
_1_ = 1.2 hrs; *a*_2_ = 0.66; 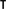
_2_ = 9.3 hrs and *c* = 0.06.

Despite this loss of fluorescence, strongly labeled somata could be readily re-identified for intervals for more than a day after *in vivo* labeling. Interestingly, we found that 8 out of 13 neurons that had been photolabeled previously could be re-labeled to fluorescence intensities between 45–85% of the intensity observed after the previous photoactivation (Figure 4E). Taken together, these experiments demonstrated that the genetically modified mice allow photolabeling of individual neurons with good signal/noise ratio for more than a day.

After photolabeling of cultured neurons expressing PA-GFP::NLS at the soma we observed an increase in fluorescence of the neurites that was likely due to diffusion of activated PA-GFP. To test to what extent this effect would also occur *in vivo* and could potentially provide morphological information we performed a series of photolabeling experiments in which we strongly and repeatedly activated the soma of individual neurons for 3 times during the period of 45 minutes (line Thy1.2#5, n = 7 neurons from 4 mice). After photolabeling we acquired image stacks of the labeled neurons and we found that this protocol lead to an intense labeling of neuronal dendrites. The label was strong enough that it could be used for anatomical tracing of neurites. We predominantly found morphologies consistent with layer 2/3 pyramidal neurons (Figure 5A, B) and only few neurons with more star-shaped arborizations that could represent putative interneurons (Figure 5C). In our hands, it was not possible to identify axons likely due to the decreasing quality of imaging conditions towards deeper layers. Furthermore, we were not able to image individual spines due to bleaching. Photolabeling could also be efficiently performed in acute brain slices prepared from mice expressing PA-GFP::NLS, which could be advantageous for targeted dendritic patching of selected neurons *in vitro* (Figure S3). Together, these findings show that PA-GFP::NLS expressing mice can provide morphological information of selected neurons that can be used for cell type identification.

**Figure 6:**
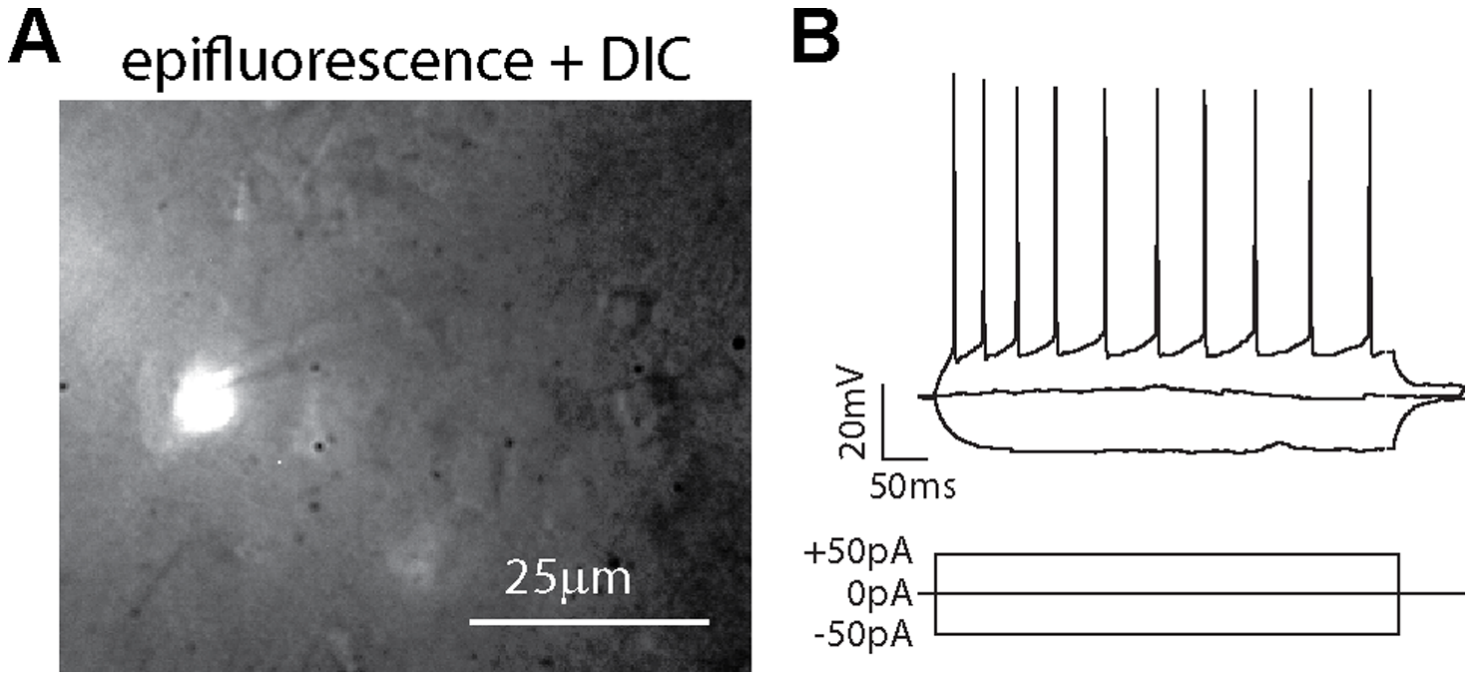
Identification of *in vivo* photolabeled neurons in the brain slice preparation. A: Composite fluorescence and DIC image of a brain slice containing a neuron previously photolabeled *in vivo* targeted with a patch pipette. B: Patch-clamp current-clamp recording of the membrane potential of the neuron shown in A in response to hyper- and depolarizing current injections.

The acute brain slice preparation provides very good experimental control and success rates for the physiological analysis of dendritic and synaptic function. We were therefore interested to test in how far *in vivo* photolabeled neurons could be re-identified in acute brain slices. We found that the photolabel persisted the preparation procedure and allowed the identification of individual neurons using epifluorescence microscopy in acute brain slices (Figure 6A). We succeeded in targeting patch-clamp whole-cell recordings to neurons that had been photolabeled and we characterized their electrophysiological properties in the current-clamp mode (Figure 6B). We found that these cells had average resting potentials of −62±7 mV and average input resistances of 266±90 MΩ, respectively (n = 5). Furthermore, the neurons displayed discharge patterns that are consistent with layer 2/3 pyramidal neurons. Our findings demonstrate that *in vivo* photolabeling can be combined with subsequent slice electrophysiology and allows the targeted patching of neurons photolabeled *in vivo*.

Furthermore, we were interested in how far the *in vivo* photolabeled neurons could be re-identified in fixed tissue. To test this, we photolabeled neurons as described above and subsequently sacrificed the mice and fixated the brains. We observed that the fixation procedure leads to a significant loss in PA-GFP fluorescence. In cell culture 60–80% of fluorescence is lost following fixation with PFA of concentrations higher than 0.07–0.18%. Fixation at low PFA concentrations leaves the tissue less stringently fixated and requires more care during slice handling. Nevertheless, individual neurons could be readily re-identified in image stacks taken from 70 µm slices of the fixed brains (Figure S4). This finding demonstrated that PA-GFP::NLS expressing mice can greatly facilitate the linkage of *in vivo* experiments on single, identified neurons and subsequent immunohistochemical analysis of their expression profile.

**Figure 7:**
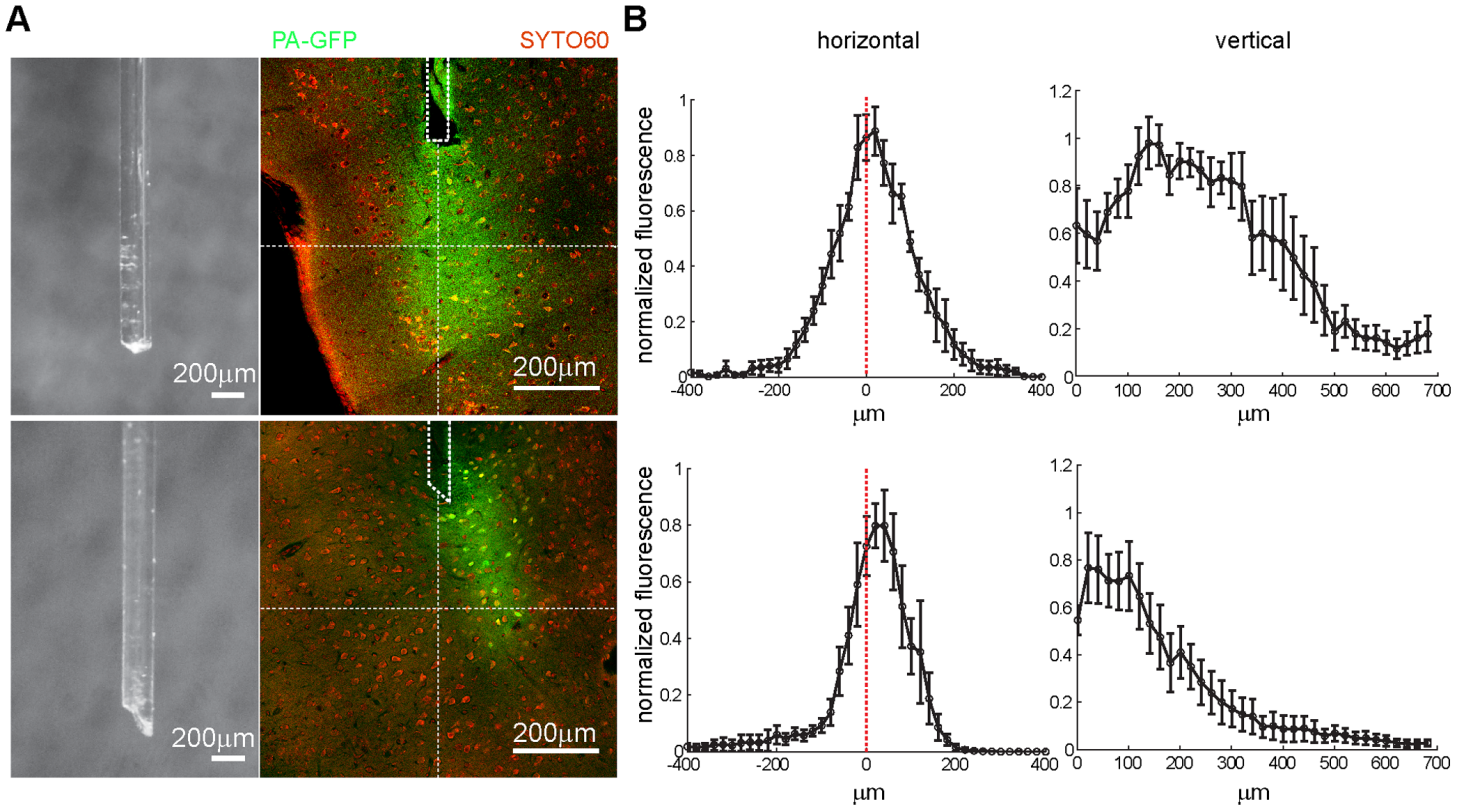
Measurement of illumination fields of optical fibers using *in vivo* photolabeling. A: Photographs of the tips of the two optical fibers used in the experiments (left, top and bottom). Confocal images of brain sections counter-stained with SYTO60 (red) with fields of photoconverted PA-GFP (green) at the end of the lesion induced by fiber implantation (dotted white lines) (right, top and bottom). Cross-like horizontal lines indicate location of fluorescence measurements for line plots. B: Line plots of averaged normalized fluorescence along the horizontal and vertical lines shown in (A). Data from 6 mice implanted with the fiber with a flat tip and 5 mice implanted with beveled tip fiber. Error bars represent SEM.

In recent years optogenetic manipulation of neuronal activity has become a valuable tool in neuroscience. Typically, optical fibers are implanted that allow illumination of neurons expressing light-sensitive proteins such as Channelrhodopsin [43]. One crucial parameter of such an approach is the delivery of light. Our current estimates how light transverses through living, scattering tissue largely relies on relatively few experiments using *in vitro* brain slices [43], [44]. The transgenic expression of PA-GFP in our mouse lines leads to homogeneous and widespread distribution of PA-GFP in the forebrain. This opens the possibility to use *in vivo* photolabeling to directly measure the spatial extent of light illumination mediated by optical fibers. We used mice of the lines Thy1.2#6 and R26 PA-GFP::NLS crossed to the EMX-Cre line and acutely implanted optical fibers used for light-mediated stimulation targeted to the perirhinal cortex [6]. The tissue surrounding the fiber was illuminated *in vivo* for 5 min with 405 nm light and brains were subsequently histologically analyzed. We observed that PA-GFP in the tissue at the end of the lesion caused by the implanted fiber was strongly photoconverted, resulting in a lasting trace of the halo arising from the fiber during *in vivo* illumination (Figure 7). We used two fibers in our experiments that differed in the shape of the tip, one with flat tip (n = 6 mice) and another with a beveled tip (n = 5 mice). When aligning individual images to the tip of the lesions and quantifying the fluorescence in the green channel, we found that the first fiber lead to a cone-like illumination along the longitudinal axis of the fiber and the other one to a tighter slanted field of illumination off the longitudinal axis. Thus, *in vivo* photolabeling in our transgenic mouse lines allows direct measurement of the field of illumination that is arising from individual optical fibers used for optogenetic approaches and therefore can be used as a useful complement to *in vitro* estimations of light spread in nervous tissue.

### Single Cell Correlation of in vivo Activity and Endogenous c-fos Expression Levels

Spontaneous activity levels of neurons in the auditory cortex *in vivo* are highly variable and their distribution is dominated by cells with low firing rates and only a minor fraction of cells showing high firing rates [45]. A recent study demonstrated that neurons with a high Fos signal are characterized by high spontaneous firing rates [46]. Are all neurons with high *in vivo* activity levels distinguished by strong Fos expression or does this rule apply only to a subpopulation of them? To answer this question it is important to identify highly active cells first and then analyze their respective expression levels, in particular as they represent only a small fraction of the whole population. We therefore combined *in vivo* calcium imaging to characterize the firing rates in a population of neurons and subsequent immunohistochemical detection of Fos in photolabeled neurons.

However, the important question arises how stable are spontaneous firing rates *in vivo*? To address this point we bulk-loaded layer 2/3 neurons in the auditory cortex of wild-type mice with the green calcium indicator OGB1-AM, implanted a small cranial window and imaged the same neuronal populations (18 populations, 43–100 neurons each) for a period of approximately 10 minutes at two time points 1.5 hrs apart [47]. We found that spontaneous activity levels in the mouse auditory cortex were highly correlated over time (Corr. coef. = 0.78, Figure S5A). We concluded that the analysis of the spontaneous firing rate at a given time point can serve as a good indicator for the activity level over the last hours (Figure S5B), which is in the temporal range of induced Fos expression [48].

**Figure 8:**
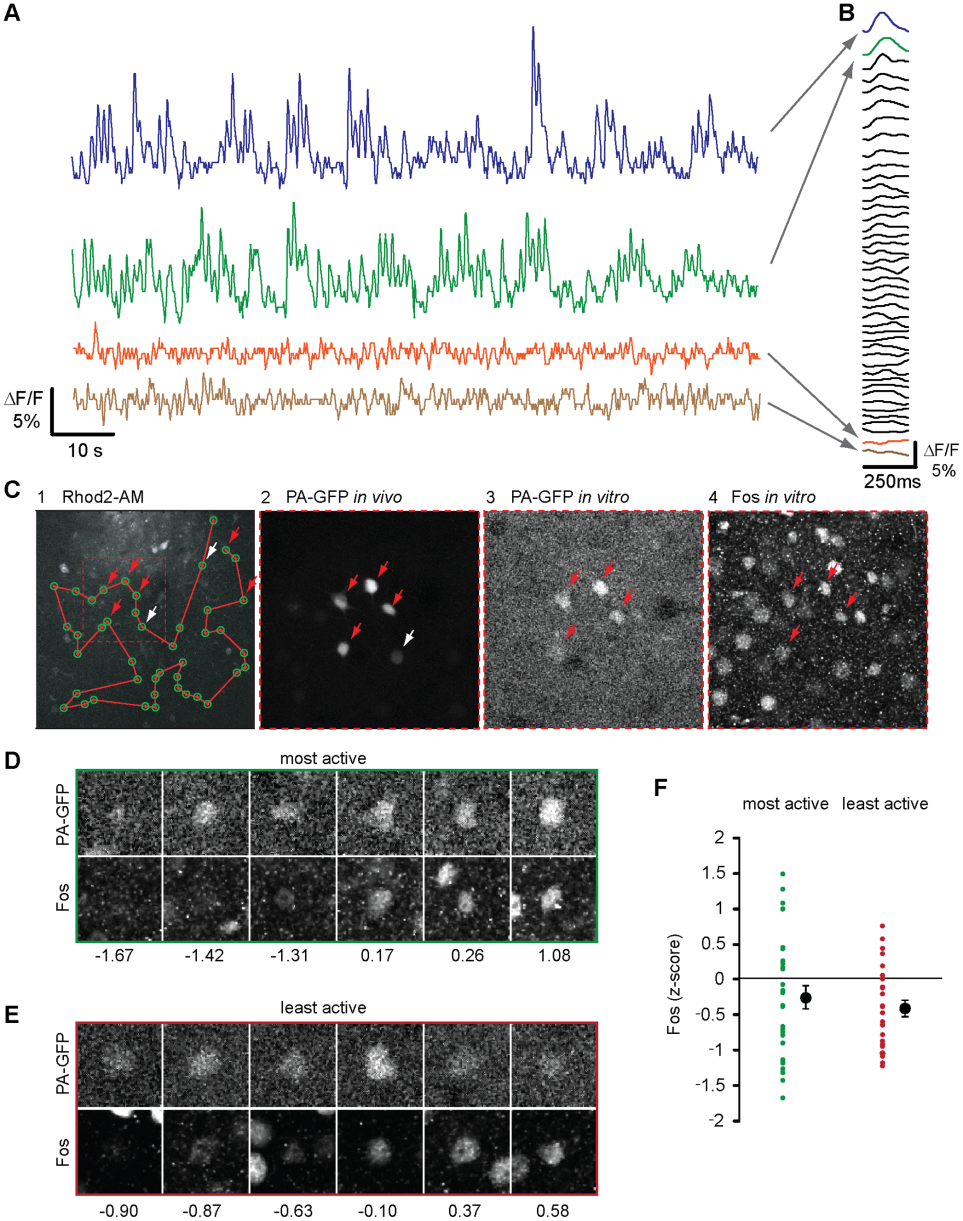
Correlation of single cell *in vivo* activity levels with Fos expression. A: Example traces of fluorescence measurements of auditory cortex neurons bulk labeled *in vivo* with the calcium indicator Rhod2. B: The fluorescence transients during spontaneously occurring population bursts were averaged for each neuron in the population and sorted by their amplitude. Arrows indicate corresponding averages for the traces shown in (A). C: Experimental workflow. 1: Calcium imaging of a neuronal population. The green circles mark simultaneously recorded neurons and the red line depicts the scan line used for fast imaging of activity. Arrows show neurons which were photolabeled following an online analysis of their activity levels. Red arrows indicate neurons re-identified *in vitro,* white arrows indicate neurons which could not be re-identified. 2: Image taken *in vivo* of neurons photolabeled after calcium imaging. 3: Same neurons shown in (2) re-identified after preparation of fixed brain slices. 4: Immunohistochemical detection of Fos in the same brain slice shown in (3). D: Examples of the PA-GFP signal and corresponding Fos label in fixed brain slices previously selected *in vivo* for high spontaneous activity levels. E: Examples of the PA-GFP signal and corresponding Fos label in fixed brain slices previously selected *in vivo* for low spontaneous activity levels. Numbers in panels D, E indicate the corresponding z-scores of Fos levels. F: Individual and average z-scores of neurons being either in the top or bottom 20% of spontaneously active neurons. Error bars represent SEM.

To combine calcium imaging with photolabeling in transgenic mice (Thy1.2#6, 9 mice) we used the red calcium indicator Rhod2-AM that allows spectral un-mixing of the PA-GFP signal. We again bulk-loaded layer 2/3 neurons with the indicator and recorded the spontaneous activity levels in a population of ∼40 neurons over 2 minutes (Figure 8A, B). Following an online analysis we chose to photolabel 20% of the neurons with either the highest or lowest activity levels in a given population (∼75% success rate). Subsequently, we fixated the brains and performed immunohistochemical detection of Fos levels in the photolabeled neurons (Figure 8C). With our staining conditions approximately 40% of the neurons showed detectable levels of Fos under basal conditions (Figure S5C). The distribution of labeling intensities for neurons was comparable to the distribution of intensities obtained from all stained cells (Figure S5D). When comparing Fos label intensities from neurons that were selected and photolabeled *in vivo* for particularly high or low spontaneous firing rates, we found in both groups considerable variability in expression levels (Figure 8D–F). We observed that neurons can have very different *c-fos* expression levels independent of their firing rates and that on the population level no significant differences were observed between highly active and weakly active neurons. On the methodological side, the association of single neuron *in vivo* firing rates and Fos detection *in vitro* was greatly facilitated by the direct labeling of the somata, which made indirect alignment of images based on landmarks obsolete.

## Discussion

In this study we generated and demonstrated the utility of genetically modified mouse lines expressing PA-GFP::NLS for various experimental approaches in which the photolabeling of neurons allows the combination of several levels of analysis on individual, identified cells. In comparison with the expression of PA-FPs using viruses, a transgenic approach makes the stereotaxic surgery for the viral injection obsolete and leads to a more even and widespread expression pattern omitting injection variability and potential infection or expression biases. A tremendous advantage of the transgenic approach is that it can be combined with tracing experiments in which the precise identity and location of tracer-labeled neurons is not known a priori. Moreover, these experiments can be carried out in early developmental stages, which are difficult to address due to difficulties in stereotaxic injections in very young animals and the delay in viral gene expression.

We believe that a major application of these mouse lines in the future will be the combination of *in vivo* calcium imaging with either histological staining or *in vitro* electrophysiology. Also, the combination of calcium imaging with intense photolabeling to reveal the dendritic morphology could be helpful to gain a better understanding of the various cell types that are forming a functional assembly in a given neuronal population. This cannot be achieved by imaging in a transgenic mouse in which only a single or few cell types are labeled. Furthermore, to understand how the transcriptional profile of a given cell translates into a specific cell type and physiological function PA-GFP::NLS expressing mice can be used to combine functional characterization *in vivo* and subsequent laser capture microdissection [49], [50] for transcriptional profiling. Mice broadly expressing photoactivatable fluorescent proteins can serve as a sensitive tool for calibration of the spatial extent of optical stimulation fields generated by optrodes or through cranial windows [51], [52]. Such straightforward monitoring is particularly useful when complex light delivery designs are applied for directed illumination. The light intensities required for photoactivation can be achieved with standard optogenetic setups, making it possible to combine optogenetic manipulations and post hoc monitoring of light delivery in the same animal.

Further variants of PA-FP expressing mice are conceivable for the future. Whereas most experimental designs are practical with a life-time of the label of one to two days, a tighter localization of the PA-FP to the nucleus could improve both label intensity and life-time. Fusion of fluorescent proteins to one of the major components of chromatin, histone 2B, is a proven strategy [14], [53]. In addition, the ongoing development of red PA-FPs [23], [54] or red calcium indicators [55] will likely expand the spectrum of fluorophores with sufficient sensitivity and signal/noise ratios for *in vivo* applications and will also offer higher flexibility in experimental approaches.

In this study we took advantage of PA-GFP::NLS expressing mice to correlate *in vivo* activity levels of individual neurons with the expression levels of the immediate early gene *c-fos* which is widely used post hoc as a bona fide marker for neuronal activity [56], [57]. Interestingly, in our experiments not all neurons with high spontaneous *in vivo* activity levels were distinguished by strong Fos expression. This result suggests that additional factors - besides neuronal activity [46] - control the level of Fos expression in individual neurons under basal conditions. The experiment nevertheless clearly demonstrated that the combination of *in vivo* imaging data with post-hoc histological analysis is greatly facilitated due to the direct labeling of individual neurons.

In this work we focused on the characterization of PA-GFP::NLS expressing mice in the context of the brain and demonstrated their applicability for several experimental approaches to elucidate neuronal functions. The R26 PA-GFP::NLS mice in particular are expected to allow broad and strong expression in most tissues of the body. We expect therefore that this mouse model can be readily used also in other biological fields in which labeling of individual cells is instrumental. Possible applications could arise in the fields of developmental biology, immunology, hematology or cancer research in which populations of cells can be labeled at a specific time point and their spread can be followed using microscopy or potentially FACS.

## Supporting Information

Figure S1 *In vitro* characterization of PA-GFP in Hek293 cells. A: Images show a Hek293 cell culture expressing PA-GFP from the CMV promoter. Upper image shows Hek293 cells before photoactivation and lower image shows the same cells after photoactivation at 750 nm. B: Two-photon emission spectrum of photoactivated PA-GFP. Hek293 cells were photoactivated at 750 nm and the fluorescence was imaged at different wavelengths ranging from 850 to 950 nm. The fluorescence was normalized to 950 nm. C: Two-photon activation spectrum of PA-GFP. Hek293 cells were activated at wavelengths ranging from 730 nm to 940 nm and fluorescence was measured at 950 nm. D: Fluorescence increase after consecutive photoactivation of PA-GFP. Hek293 cells were activated consecutively at 730 nm and the fluorescence increase was measured at 950 nm.
(TIF)

Figure S2 PA-GFP expression in the R26 PA-GFP::NLS mouse. A: Coronal sections of the neocortex obtained from the R26 PA-GFP::NLS knock-in mouse line that were immunohistochemically stained for PA-GFP. Without expression of Cre-recombinase PA-GFP::NLS expression is essentially blocked by the stop-cassette (left). Crossing this mouse line with an EMX1-Cre mouse line leads to the removal of the Stop cassette and strong expression of PA-GFP::NLS can be detected (right). B: *In vivo* imaging in the auditory cortex of the R26 PA-GFP::NLS mouse line crossed with a Nestin-Cre mouse line. Neurons were photolabeled in a square shaped ROI.
(TIF)

Figure S3 Photolabeling of neurons in acute brain slices. Maximum intensity projection of a two-photon image stack taken from two neurons that had been previously photolabeled *in vitro*. Details of the neuronal morphology can be visualized by the diffusion of PA-GFP from the soma, the site of photoactivation, into neurites. Red arrows show putative axon. Note that neurons at the surface of the acute brain slice that were damaged by the cutting procedure can show high levels of autofluorescence.
(TIF)

Figure S4 Re-identification of neurons in fixed brain slices that had been previously photolabeled i*n vivo.* The rows correspond to three examples. The first column shows an epifluorescence image taken *in vivo*. Individual neurons are indicated by red arrows. The middle column shows two-photon *in vivo* images of neurons that had been photolabeled in an arbitrary pattern. The right column shows two-photon images of the same neurons in a brain slice after fixation.
(TIF)

Figure S5 Stability of spontaneous activity levels *in vivo* and Fos levels in immunohistochemically identified neurons. A: Populations of neurons in the auditory cortex *in vivo* were bulk loaded with the calcium sensitive dye OGB1 and the levels of spontaneous activity were measured for approximately 10 minutes at two time points (t = 0 min, t = +90 min). To quantify spontaneous activity, we measured the average change in fluorescence (*ΔF/F*) during a spontaneously occurring population burst for each neuron. In the scatter plot, data for individual neurons is shown (18 populations, 43–100 neurons each). The activity levels between both time points over one hour apart are strongly correlated. B: Quantification of the fraction of neurons that have been in the 20% most or 20% least active neurons in a given imaged population at time point t = 0 min, that also fall in the same quantile at time point t = 90 min. Individual dots represent data per imaged neuronal population. Error bars represent SD. C: Brain slices were stained for the neuronal marker NeuN and for Fos. The Venn diagram shows the amount of NeuN (NeuN^+^) and Fos (Fos^+^) and double-positive cells (NeuN^+^+Fos^+^). Approximately 40% of all neurons show detectable Fos levels. D: Histogram of the distribution of the z-scores of Fos positive and double positive neurons. The distribution of Fos levels obtained from all cells in an image plane (Fos^+^) is comparable to the distribution of Fos levels in neurons only (NeuN^+^+Fos^+^). This shows that the Fos levels measured from all cells in an image plane serve well as an estimate of the distribution of Fos levels in neurons and can be used to construct z-scores for neurons.
(TIF)
