# Supplementary figures and images for: Transgenic Mouse Models Enabling Photolabeling of Individual Neurons In Vivo
Source: PLoS One. 2013 Apr 23;8(4):e62132. doi: 10.1371/journal.pone.0062132 (PMC3633923; doi:10.1371/journal.pone.0062132)

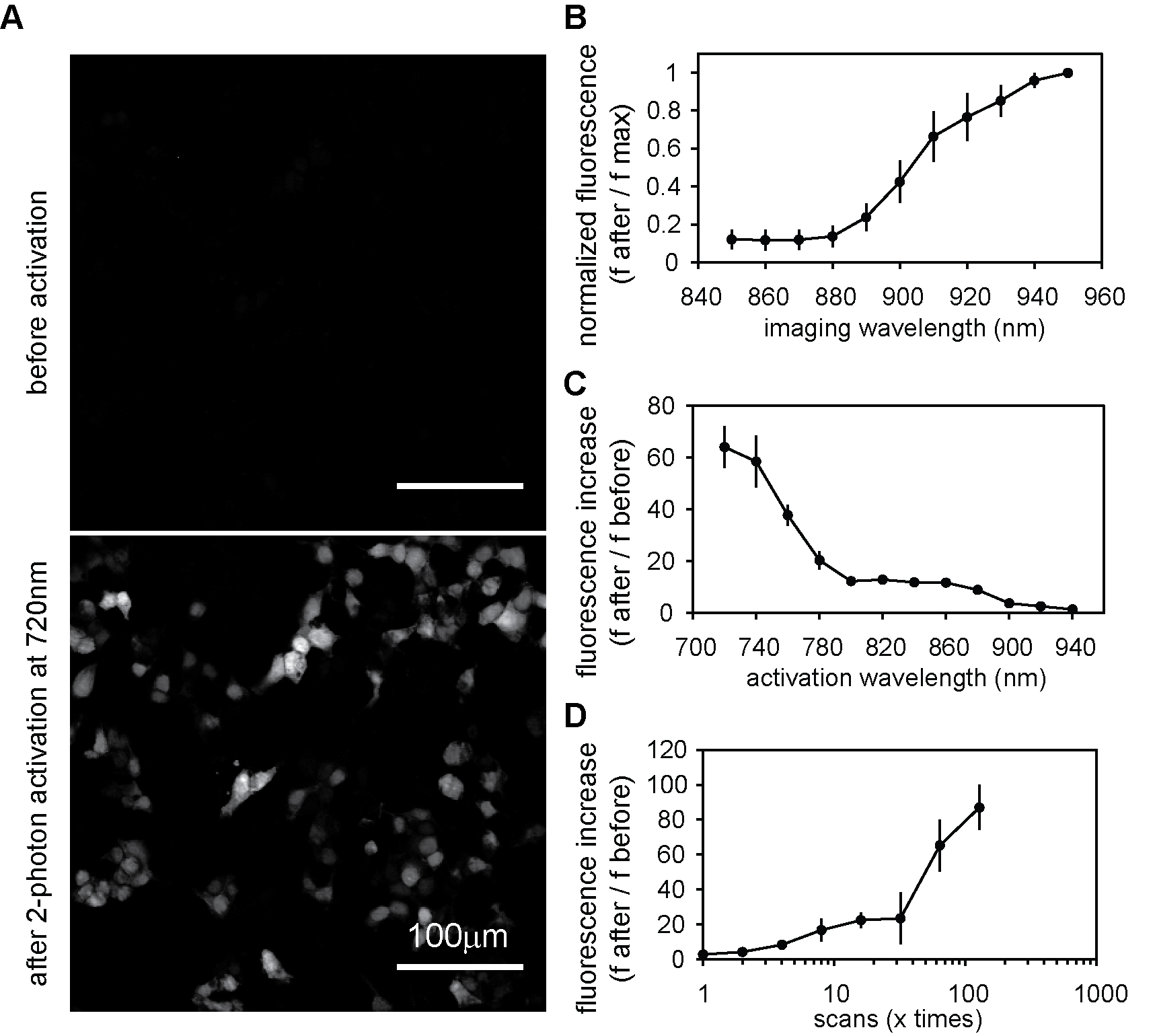

Supplement: Figure S1 — In vitro characterization of PA-GFP in Hek293 cells. A: Images show a Hek293 cell culture expressing PA-GFP from the CMV promoter. Upper image shows Hek293 cells before photoactivation and lower image shows the same cells after photoactivation at 750 nm. B: Two-photon emission spectrum of photoactivated PA-GFP. Hek293 cells were photoactivated at 750 nm and the fluorescence was imaged at different wavelengths ranging from 850 to 950 nm. The fluorescence was normalized to 950 nm. C: Two-photon activation spectrum of PA-GFP. Hek293 cells were activated at wavelengths ranging from 730 nm to 940 nm and fluorescence was measured at 950 nm. D: Fluorescence increase after consecutive photoactivation of PA-GFP. Hek293 cells were activated consecutively at 730 nm and the fluorescence increase was measured at 950 nm. (TIF) [file pone.0062132.s001.tif]

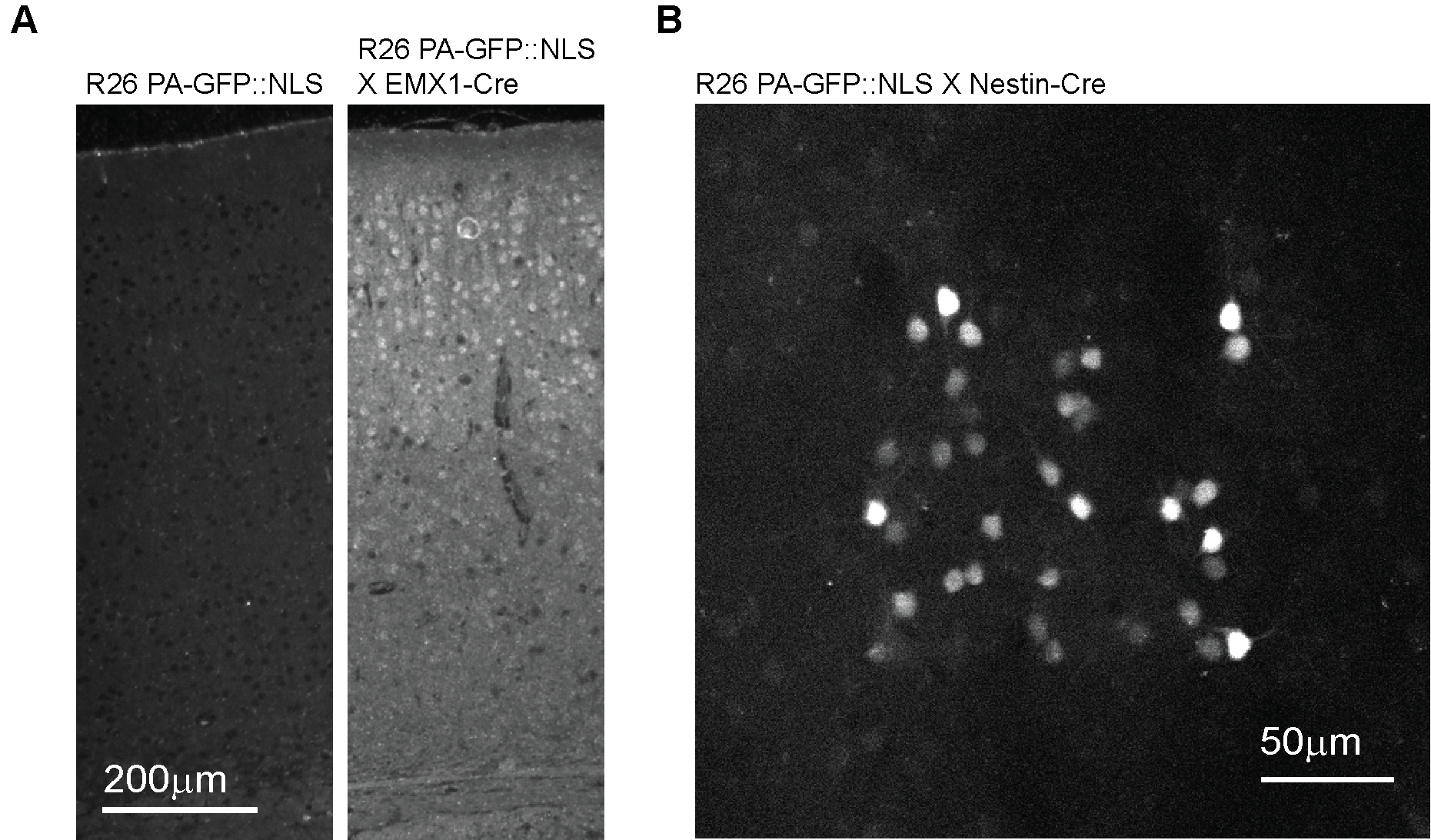

Supplement: Figure S2 — PA-GFP expression in the R26 PA-GFP::NLS mouse. A: Coronal sections of the neocortex obtained from the R26 PA-GFP::NLS knock-in mouse line that were immunohistochemically stained for PA-GFP. Without expression of Cre-recombinase PA-GFP::NLS expression is essentially blocked by the stop-cassette (left). Crossing this mouse line with an EMX1-Cre mouse line leads to the removal of the Stop cassette and strong expression of PA-GFP::NLS can be detected (right). B: In vivo imaging in the auditory cortex of the R26 PA-GFP::NLS mouse line crossed with a Nestin-Cre mouse line. Neurons were photolabeled in a square shaped ROI. (TIF) [file pone.0062132.s002.tif]

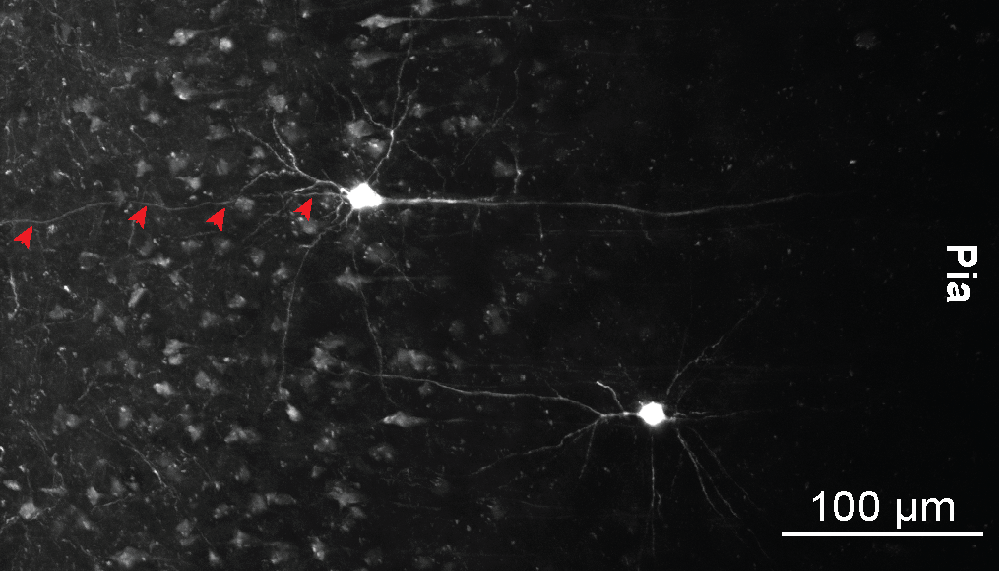

Supplement: Figure S3 — Photolabeling of neurons in acute brain slices. Maximum intensity projection of a two-photon image stack taken from two neurons that had been previously photolabeled in vitro. Details of the neuronal morphology can be visualized by the diffusion of PA-GFP from the soma, the site of photoactivation, into neurites. Red arrows show putative axon. Note that neurons at the surface of the acute brain slice that were damaged by the cutting procedure can show high levels of autofluorescence. (TIF) [file pone.0062132.s003.tif]

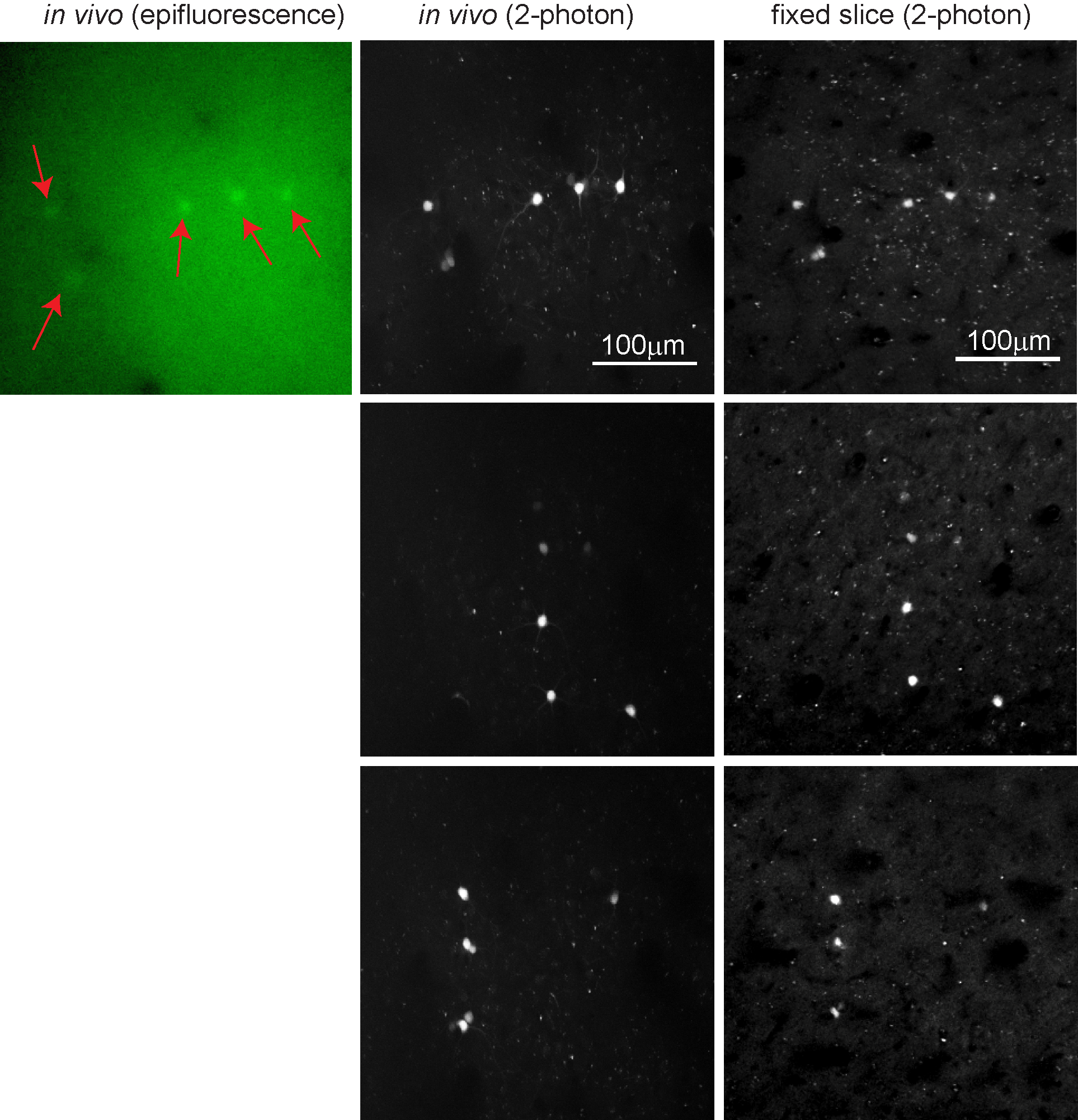

Supplement: Figure S4 — Re-identification of neurons in fixed brain slices that had been previously photolabeled in vivo. The rows correspond to three examples. The first column shows an epifluorescence image taken in vivo. Individual neurons are indicated by red arrows. The middle column shows two-photon in vivo images of neurons that had been photolabeled in an arbitrary pattern. The right column shows two-photon images of the same neurons in a brain slice after fixation. (TIF) [file pone.0062132.s004.tif]

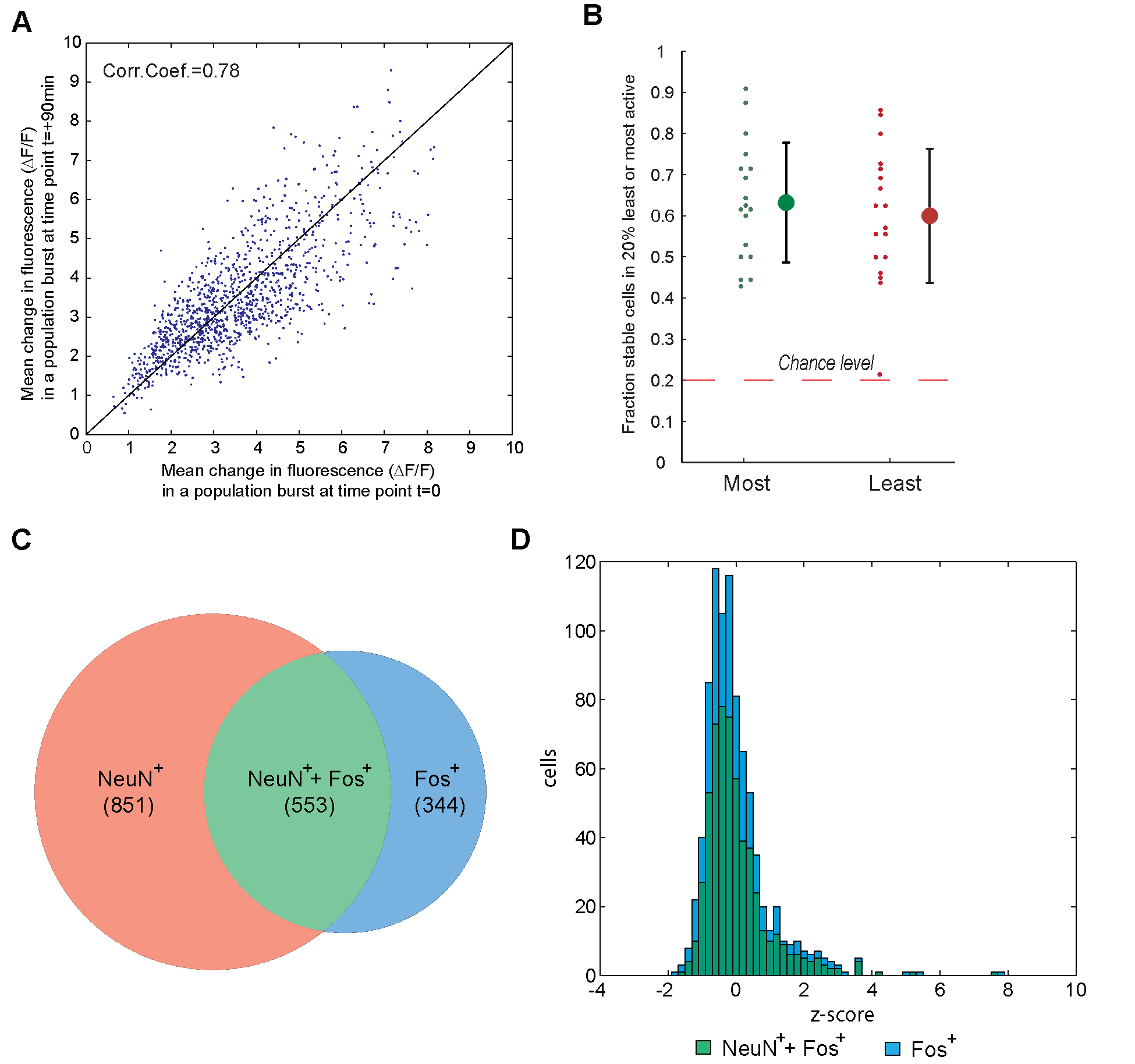

Supplement: Figure S5 — Stability of spontaneous activity levels in vivo and Fos levels in immunohistochemically identified neurons. A: Populations of neurons in the auditory cortex in vivo were bulk loaded with the calcium sensitive dye OGB1 and the levels of spontaneous activity were measured for approximately 10 minutes at two time points (t = 0 min, t = +90 min). To quantify spontaneous activity, we measured the average change in fluorescence (ΔF/F) during a spontaneously occurring population burst for each neuron. In the scatter plot, data for individual neurons is shown (18 populations, 43–100 neurons each). The activity levels between both time points over one hour apart are strongly correlated. B: Quantification of the fraction of neurons that have been in the 20% most or 20% least active neurons in a given imaged population at time point t = 0 min, that also fall in the same quantile at time point t = 90 min. Individual dots represent data per imaged neuronal population. Error bars represent SD. C: Brain slices were stained for the neuronal marker NeuN and for Fos. The Venn diagram shows the amount of NeuN (NeuN+) and Fos (Fos+) and double-positive cells (NeuN++Fos+). Approximately 40% of all neurons show detectable Fos levels. D: Histogram of the distribution of the z-scores of Fos positive and double positive neurons. The distribution of Fos levels obtained from all cells in an image plane (Fos+) is comparable to the distribution of Fos levels in neurons only (NeuN++Fos+). This shows that the Fos levels measured from all cells in an image plane serve well as an estimate of the distribution of Fos levels in neurons and can be used to construct z-scores for neurons. (TIF) [file pone.0062132.s005.tif]
